# WIsH: who is the host? Predicting prokaryotic hosts from metagenomic phage contigs

**DOI:** 10.1093/bioinformatics/btx383

**Published:** 2017-07-13

**Authors:** Clovis Galiez, Matthias Siebert, François Enault, Jonathan Vincent, Johannes Söding

**Affiliations:** 1 Quantitative and Computational Biology Group, Max-Planck Institute for Biophysical Chemistry, 37077 Göttingen, Germany; 2 Université Clermont Auvergne, CNRS, LMGE, Clermont-Ferrand, France

## Abstract

**Summary:**

WIsH predicts prokaryotic hosts of phages from their genomic sequences. It achieves 63% mean accuracy when predicting the host genus among 20 genera for 3 kbp-long phage contigs. Over the best current tool, WisH shows much improved accuracy on phage sequences of a few kbp length and runs hundreds of times faster, making it suited for metagenomics studies.

**Availability and implementation:**

OpenMP-parallelized GPL-licensed C ++ code available at https://github.com/soedinglab/wish.

**Supplementary information:**

Supplementary data are available at *Bioinformatics* online.

## 1 Introduction

Viruses are key components of almost all known ecosystems (Edwards and Rohwer, 2005). They regulate biological diversity in various environments from oceans to the human gut by depleting dominant species (De Paepe *et al.*, 2014; Lehahn *et al.*, 2014) and are even estimated to be responsible for the death of 20% of the living ocean biomass per day (Suttle, 2007). Viruses are therefore central for understanding microbial ecology and dynamics.

Even though phages (i.e. viruses infecting bacteria and archaea) represent the majority of the global virosphere, their comprehensive study has been hampered by the necessity of isolating and cultivating their host. Viral metagenomics circumvent this limitation, increasingly unveiling new viral genomic sequences from a wide range of environments (Bolduc *et al.*, 2016; Edwards and Rohwer, 2005). As a drawback, the identity of the hosts remains unknown for these newly discovered viruses, limiting our ecological understanding of the microbiome. Different methods exist to predict prokaryotic hosts for phage sequences in metagenomes, based either on co-abundance, sequence homology, similarity to other phages (Villarroel *et al.*, 2016) or sequence composition similarity between viruses and their hosts (Edwards *et al.*, 2016).

Among tools taking this last approach, VirHostMatcher (Ahlgren *et al.*, 2016) has reported the best accuracy (proportion of correct predictions) on full-length viral genomes: between 33 and 64% at the genus level depending on the dataset. But its performance drops notably for shorter sequences, falling by 36% at 5 kbp length.

However, contigs of a few kbp length are common in viral metagenomic data due to shallow coverage and intra-population variation (Smits *et al.*, 2014). In addition, the running time of VirHostMatcher hinders its use on large datasets (Supplementary Table S5). Here we introduce WIsH, a tool to predict the prokaryotic host of viral contigs with good accuracy for contigs as short as 3 kbp that runs several hundred times faster than VirHostMatcher.

## 2 Materials and methods

The estimated *k*-mer frequencies classically used for host prediction using genomic composition become very noisy for short phage contigs. We therefore adopted a suited probabilistic approach. First, we train a homogeneous Markov model of order 8 (Supplementary Fig. S2) for each potential host genome (WIsH -c build -g prokaryoteGenomesDir -m modelDir). We then compute the likelihood of a contig under each of the trained Markov models (WIsH -c predict -g phageContigsDir -m modelDir -r outputResultDir) and predict *de novo* (i.e. without relying on any known phage-host interaction) the host whose model yields the highest likelihood (details in Supplementary Material).

To evaluate the performance of WIsH an VirHostMatcher, we used the 3780 full prokaryotic genomes of the KEGG database (Kanehisa *et al.*, 2017) and the 1420 phages in the RefSeq Virus database (Brister *et al.*, 2015) for which a host was annotated in this database.

WIsH can compute *P*-values when provided with the parameters of the Gaussian null-distributions of each Markov model (option -n KeggGaussianFits.tsv -b). The Gaussian parameters were precomputed for each model as explained in Supplementary Material Section S1.2.

## 3 Results

**Fig. 1:**
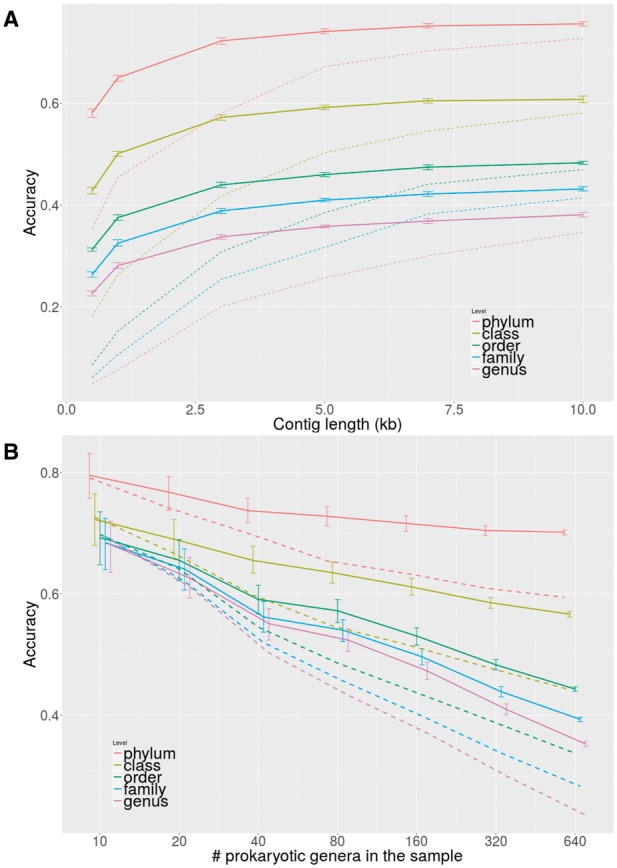
Solid lines for WIsH (errors bars showing 95% confidence interval) and dashed lines for VirHostMatcher. (**A**) Prediction accuracy over phage contig length for 3780 potential bacterial and archaeal host genomes from 965 genera. (**B**) Accuracy for 3 kbp phage contigs for various numbers of prokaryotic host genera per sample, estimated by randomly drawing (300 replications) potential hosts from the indicated number of genera

WIsH outperforms VirHostMatcher at every taxonomic level (Fig. 1A, and ROC curves in Supplementary Fig. S4). Although the accuracy for long contigs is improved only by a few percentage points, predictions for contigs of 3 kB have 60% higher accuracy than those of VirHostMatcher. Similar results were obtained on the original VirHostMatcher benchmark set (Ahlgren *et al.*, 2016) (Supplementary Table S1). At a *P*-value threshold of 0.06, WIsH predicts hosts for 50% of the phage sequences with 75% accuracy at the family level (Supplementary Fig. S1). Furthermore, these accuracies can be considered as lower bonds as in practice the user can restrict the set of host genomes to those actually present in the sample. For contigs of length 3 kbp, WisH accuracy reaches 63% for 20 potential host genera per sample and 52% for 80 genera per sample (Fig. 1B).

Paez-Espino *et al.* (2016) describe a set of 125,842 metagenomic viral contigs (mVCs) of 11 kbp median length from various environments. The original host prediction mainly used CRISPR and t-RNA sequence matches and made predictions for only 7.7% of the mVCs. With a *P*-value threshold of 0.1 WIsH annotated 59% of the mVCs and the predicted host families matched the previous annotation in 70% of the cases, giving a lower bound on the accuracy (Supplementary Fig. S10).

Runtime measurements of WIsH on a 16-core 2.60GHz Intel Xeon yielded a speed of 55 kbp/s, several hundred times faster than VirHostMatcher (Supplementary Table S5).

Prokaryotic taxonomy usually follows subjective, historic criteria that can differ markedly among phyla, limiting the observed prediction accuracies. Using the fraction of identical nucleotides in 16S rRNA genes as quantitative measure of evolutionary relatedness (Yarza *et al.*, 2014), accuracies improve drastically, e.g. from 47 to 63% on the family level when using the full set of 3780 host reference genomes (Supplementary Table S3).

The phages that show the poorest predictions tend to have longer genomes and to encode more tRNA (Supplementary Material Section S7.1 and Supplementary Figs S11–S14). These phages may be more independent from their hosts and may have less selective pressure to adapt their genomes to their hosts.

## 4 Conclusion

WIsH predicts hosts for short phage sequences with a good accuracy and very high speed. We hope that it will help in the investigation of microbial ecology through metagenomics shotgun sequencing of microbiomes.

## Supplementary Material

Supplementary Materials
