# Supplementary material for: WIsH: who is the host? Predicting prokaryotic hosts from metagenomic phage contigs
Source: Bioinformatics. 2017 Jul 13;33(19):3113–4. doi: 10.1093/bioinformatics/btx383 (PMC5870724; doi:10.1093/bioinformatics/btx383)
Supplement: Supplementary Materials [file suppl_clean_btx383.pdf]

Supplementary materials

June 8, 2017

1 Theoretical background

1.1 Markov modeling of host genomes

For a potential bacterial or archaeal host H, a Markov model of order k is trained on its genome by a maximum-likelihood approach. The maximum likelihoods solution of the Markov model's conditional probability of observing nucleotide x_{k+1} given preceding nucleotides $x_1...x_{k+1}$ is

$$p_H(x_{k+1}|x_1...x_k) = \frac{\#x_1 \dots x_{k+1} \text{ in H} + 0.25\alpha}{\#x_1 \dots x_k \text{ in H} + \alpha}\,, \tag{1}$$

where $\#x_1...x_{k+1}$ is the number of times that the string $x_1 \dots x_{k+1}$ has been observed in the genome of H and α is a pseudo-count parameter.

The log-likelihood per position for a sequence $\mathbf{y} = y_1...y_N$ of a phage Φ under the model H is:

$$\text{LL}(\Phi|H) = \frac{1}{N-k}\sum_{i=1}^{N-k} \log p_H(y_{i+k}|y_i...y_{i+k-1}) \tag{2}$$

WIsH returns a matrix that contains in its cell (i, j) the mean log-likelihood $\text{LL}(\Phi_j|H_i)$ of the phage sequence Φ_j under the model H_i in the training set.

1.2 p-values

If the phage ϕ does not infect the prokaryotic host H, we assume that composition of their genomes will be independent. Then $\text{LL}(\Phi|H)$ will be an average of independent identically distributed random variables $\log p_H(y_{i+k}|y_i...y_{i+k-1})$, thus following a Gaussian distribution by the central limit theorem.

For a given host H, by selecting phage sequences $\bar{\Phi}_1, ...\bar{\Phi}_n$ that do not infect H, one can fit by maximum-likelihood the parameters μ_H, σ_H of the Gaussian null-distribution for H:

$$\mu_H = \frac{1}{n}\sum_{i=1}^{n} \text{LL}(\bar{\Phi}_i|H) \tag{3}$$

$$\sigma_H^2 = \frac{1}{n}\sum_{i=1}^{n} \text{LL}(\bar{\Phi}_i|H)^2 - \mu_H^2 \tag{4}$$

If an organism H is predicted to be the host of a phage Φ with a score of $s = \text{LL}(\Phi|H)$, then we can compute the following p-value:

$$p = P(S \geq s) \tag{5}$$

where $S \sim \mathcal{N}(\mu_H, \sigma_H)$.

2 Benchmark details

2.1 Building new null-models

We provide in the file KeggGaussianFits.tsv the pre-computed parameters for the Gaussian null-distribution of every prokaryotic genome used in the benchmark. If a user wants to compute the parameters for the null-distribution for another prokaryotic genome *newProk* of genus G, the following process can be used:

- Get a (large) set Z of phages that are known to infect another genus than G,
- build a model with WIsH using the genome file **newProk.fna**,
- run the WIsH prediction of the model of *newProk* against the set Z,
- fit (*e.g.* using maximum likelihood) a gaussian distribution over the scores given in the **likelihood.matrix** file,
- add a line to a file **negFits.tsv** with the following format:
  newProk<TAB>Mean<TAB>StandardDeviation

To get the p-values associated to a prediction, the user can then use the options *-b -n negFits.tsv* when running a prediction.

2.2 p-value and accuracy

We define the precision as the fraction of correct predictions among all predictions:

$$\text{Precision} = \frac{TP}{TP + FP} \tag{6}$$

We moreover define the ”fraction predicted” as:

$$\text{Fraction predicted} = \frac{TP + FP}{TP + FP + TN + FN} \tag{7}$$

The *accuracy* is defined as the precision when making prediction on all the phage sequences (*i.e.*precision for a predicted fraction of 1.0). Figure 1 shows the precision and the fraction of predictions which the user can expect for different p-values. The user can fix a suited threshold depending on whether a low false discovery rate or a high amount of predictions is desired.

2.3 Influence of the order of the Markov model

As shown the figure 2, the accuracy is generally maximal for order 8. The drop in accuracy for higher orders could be explained by the too high specificity of the models to their host, and fail at detecting a strong signal in more distant genomes such as phages that infect them. Another point is that at order 9 the model parameters cannot be estimated accurately enough anymore, because a genome of typical length 5 Mbp will yield only about $5.10^6/4^{10} = 4.8$ counts per 10-mer, but will still yield 19 counts per 9-mer. The optimal order $k = 8$ is thus a general compromise learning as complex a model as possible with conditional probabilities that are still sufficiently reliably estimated.

The order of the model can be tuned accordingly to the size of the prokaryotic genome use for training. Figure 3 shows the accuracies of the prediction depending on the order of the Markov model when training on truncated versions of the prokaryotic genomes (down to 25kbp and 1Mbp).

2.4 ROC curve on WIsH benchmark

For plotting the ROC curves in figure 4, z-scores were used instead of raw log-likelihood (calling WIsH with `-z` option) to have comparable values between two different phages. As for log-likelihood values, the higher the z-score, the more probable the interaction is. The areas under the ROC curve are comparable when using WIsH or VirHostMatcher for full-genome phage sequences, although slightly better for WIsH.

2.5 Detailed accuracies on different benchmarks

Dataset	# Viral sequences	# Prokaryotic genomes	Contig size	WIsH	VirHostMatcher ($k = 6$)
WIsH benchmark	1 420	3 780	1kb	**28.0**	7.5
			5kb	**35.5**	25.6
			10kb	**38.7**	34.8
			Full genome	**42.5**	42.3
VHM benchmark	1 427	31 986	1kb	**28.2**	$\approx$ 7 *
			5kb	**32.8**	$\approx$ 21 *
			10kb	**33.9**	$\approx$ 26 *
			Full genome	**35.3**	33 **

*From (Ahlgren *et al.*, 2016), Fig3A.
**From (Ahlgren *et al.*, 2016), Table 3.

Table 1: Accuracies at genus level.

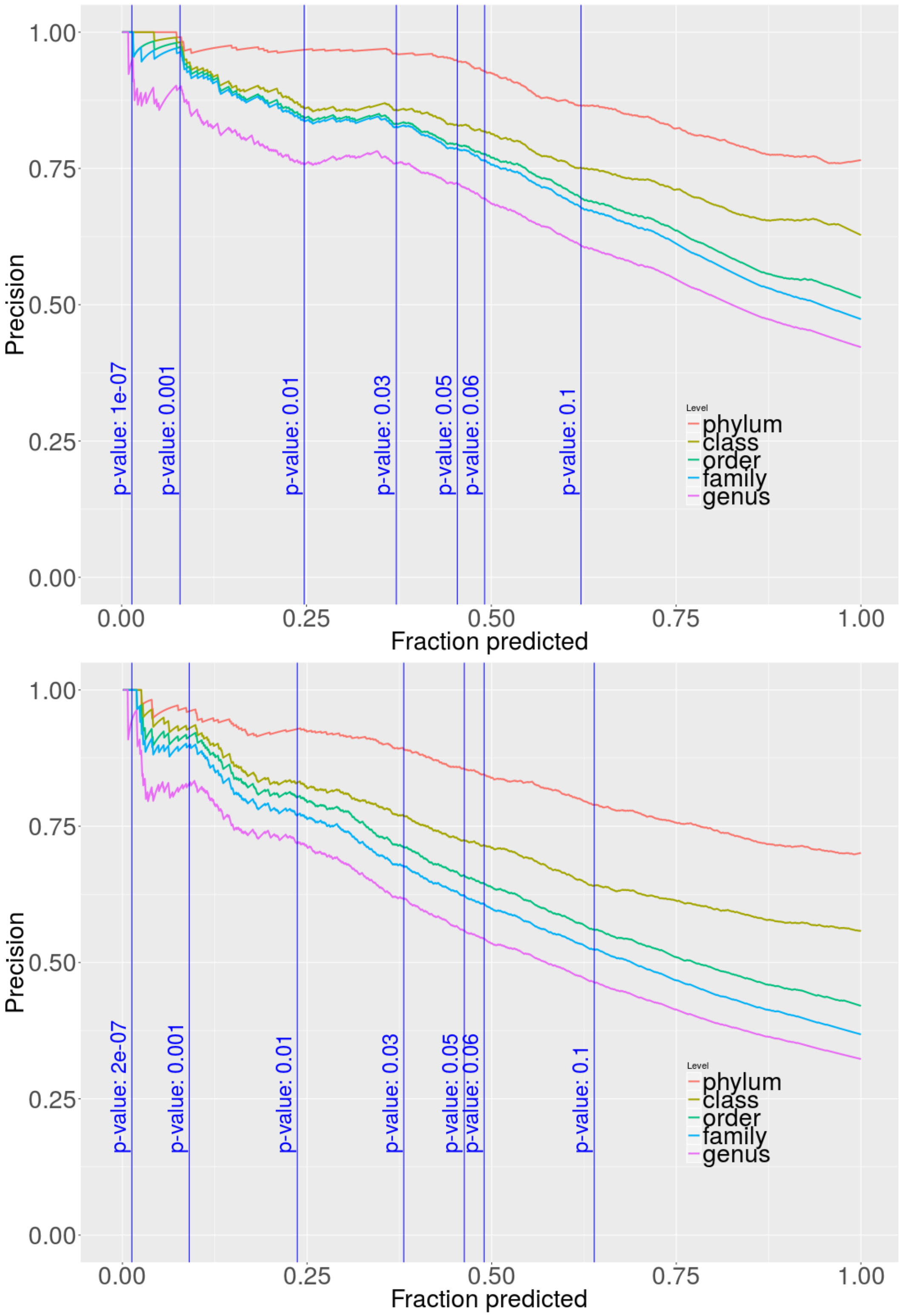

Figure 1: Depending on the desired trade-off between number of predicted sequences and accuracy of the predictions, the user can select a suitable p-value threshold. **Top**: Evaluation on full-length phage genomes. **Bottom**: Evaluation on 3kbp contigs.

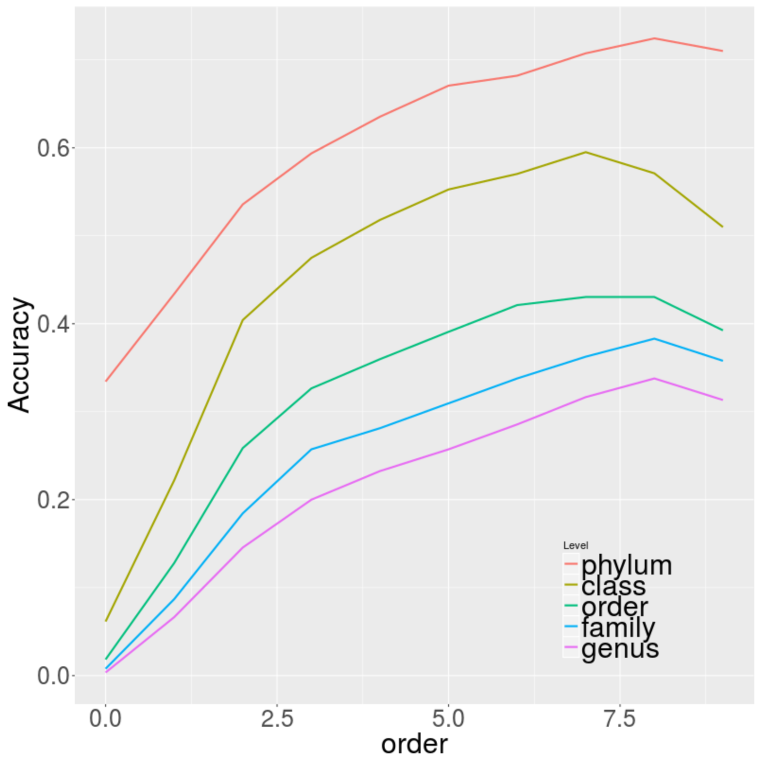

Figure 2: Accuracies of the host prediction on 3 kbp contigs, depending on the order of the Markov model.

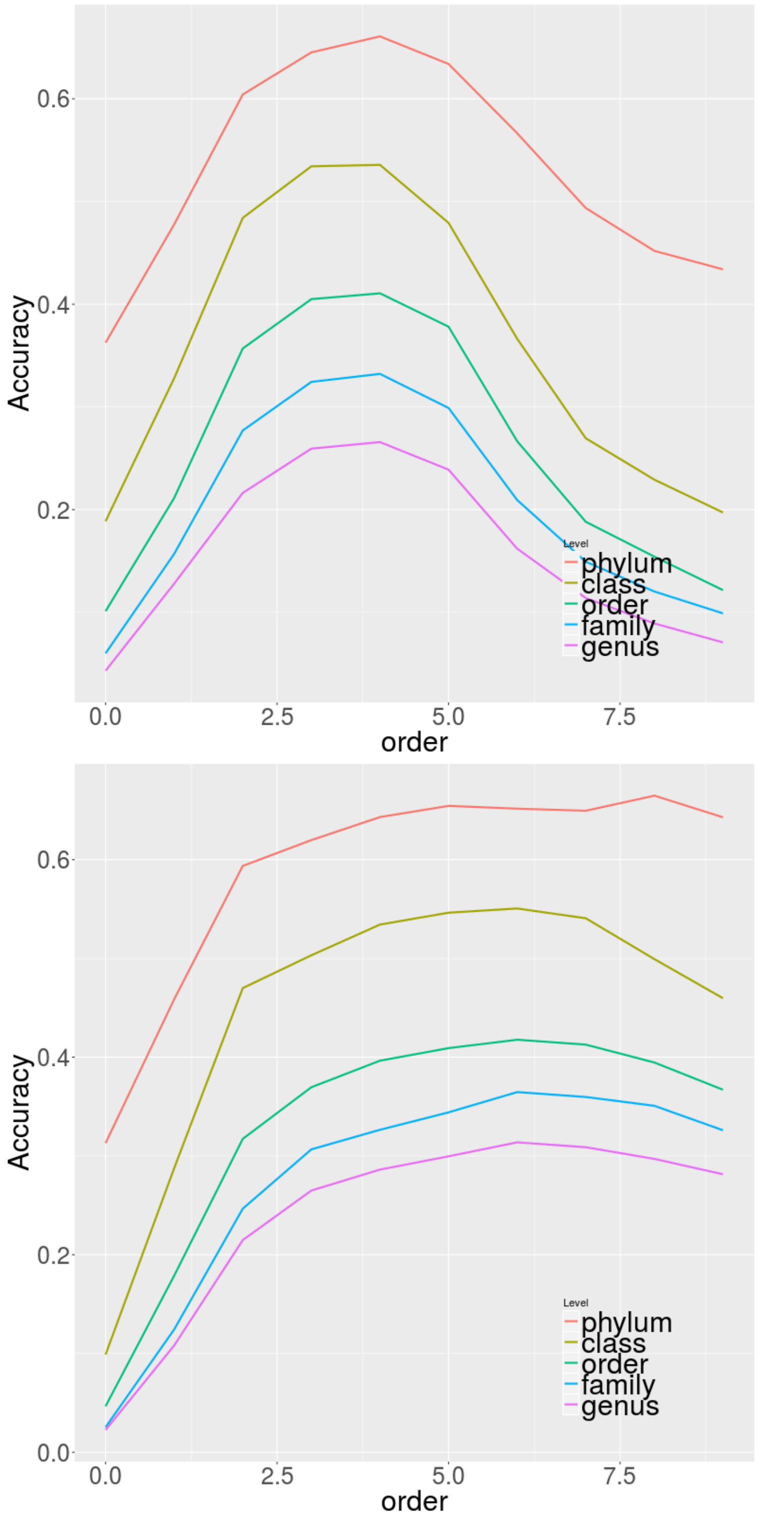

Figure 3: Accuracies of the host prediction on 3 kbp contigs, depending on the order of the Markov model. **Top**: Training prokaryotic genomes truncated to 25kbp. **Bottom:** Training prokaryotic genomes truncated to 1Mbp.

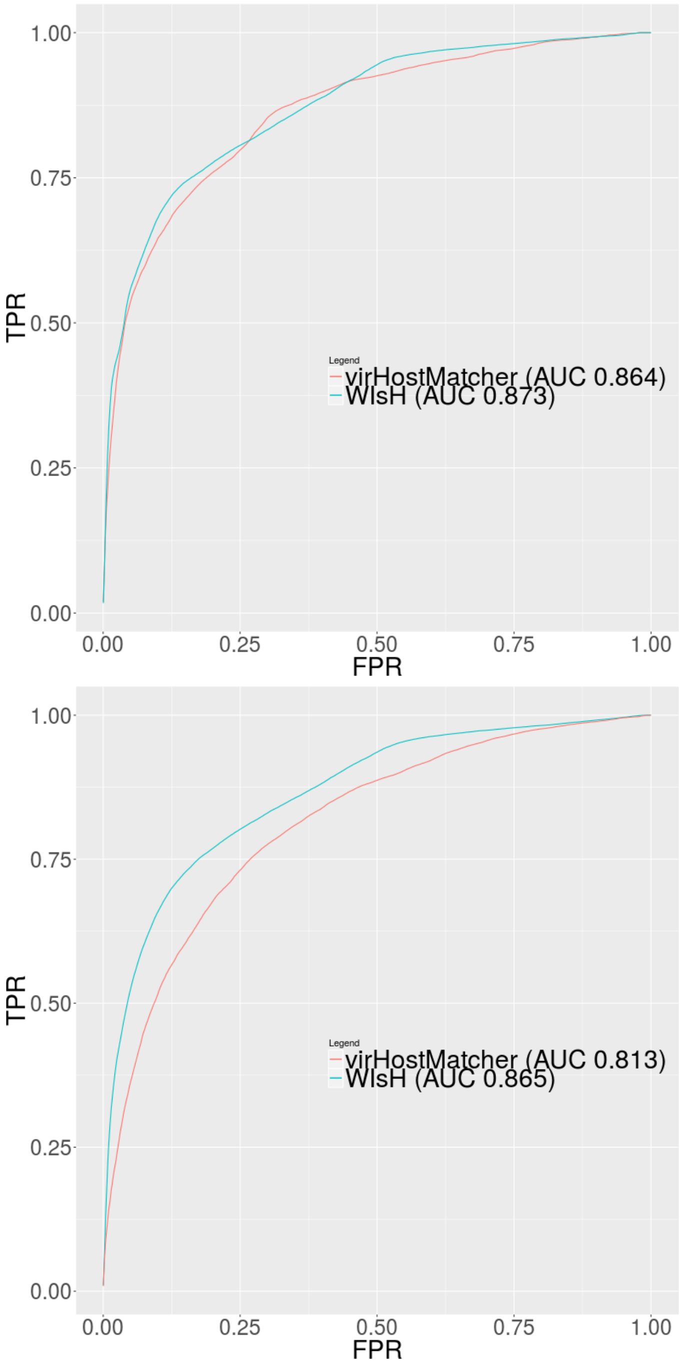

Figure 4: Receiver operator (ROC) curves for all predictions ranked by z-scores. TPR = TP / (TP + FN) = true positive rate, FPR = FP / (FP + TN) = false positive rate. AUC stands for Area Under the ROC Curve. **Top**. ROC for predicting on full-length phage genomes. **Bottom**. ROC when predicting on 3 kpb contigs.

3 Evaluation under lower prokaryotic diversity

In real use cases, the genera richness of the prokaryotic fraction is typically between 10 and 150 genera (Santigli *et al.*, 2016; França *et al.*, 2016; Nasidze *et al.*, 2009). If the prokaryotic fraction has been sequenced as well, one can restrict the prediction of the host to the genera that are present (or even dominant) in the prokaryotic fraction. By randomly subsampling fewer genus (respectively orders), Figure 5 (respectively Figure 6) present an estimate of the expected accuracy when restricting the prediction to the corresponding number of genus (resp. orders).

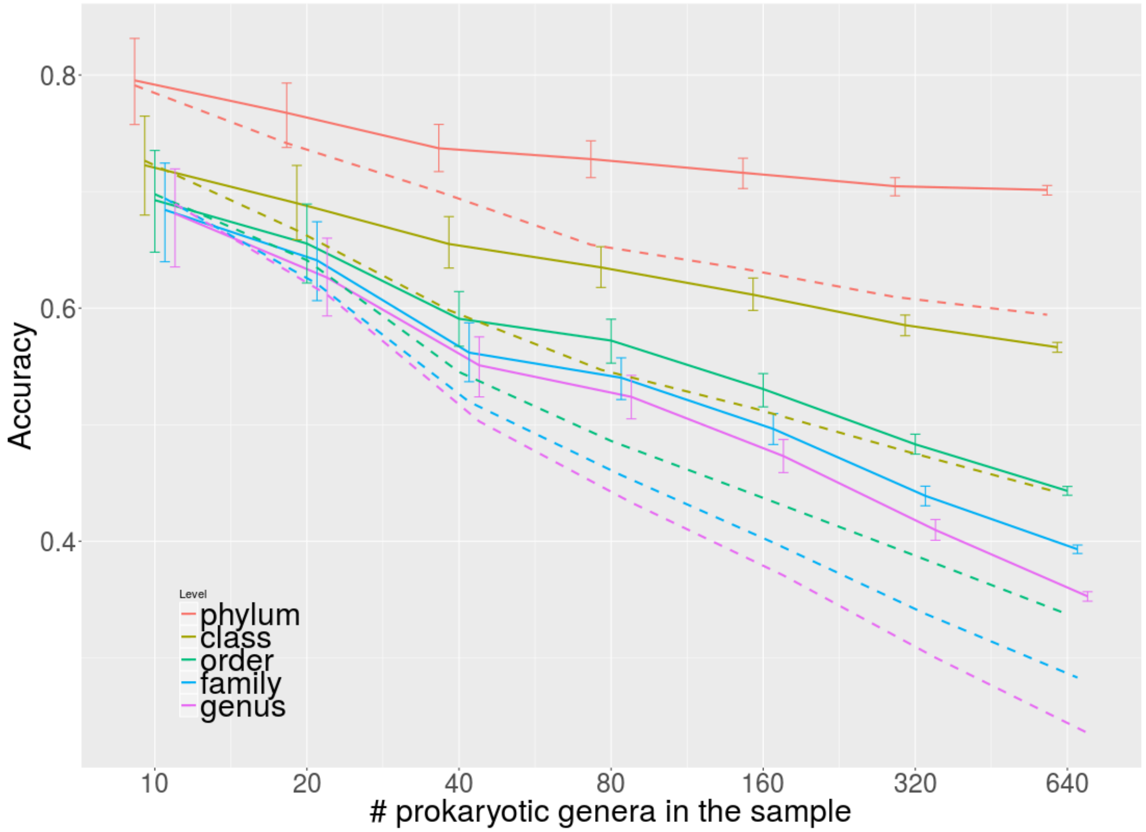

Figure 5: Expected accuracy when predicting on 3 kbp phage sequences for hosts belonging to fewer genus. The error bars indicate 95% of confidence interval, over 300 replicates. For a better readability of the plot, the error bars are horizontally shifted by 0.1. Solid lines: WisH, dashed lines: VirHostMatcher.

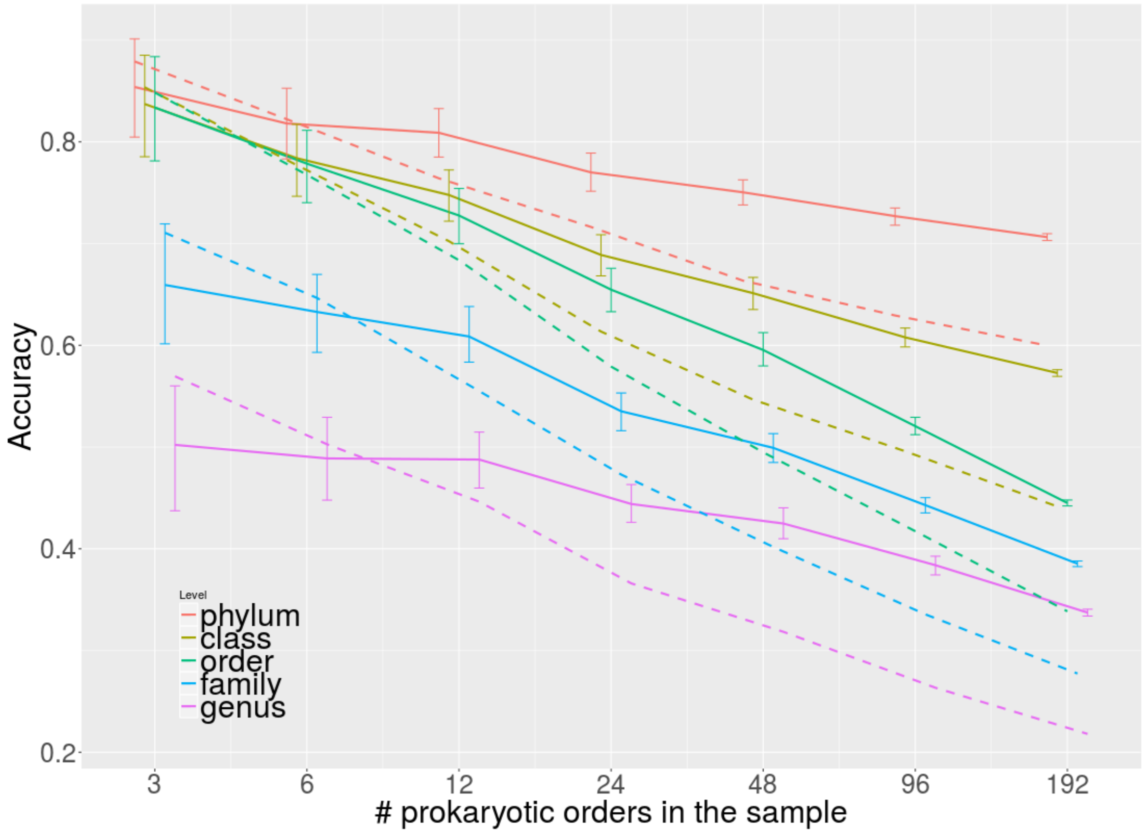

Figure 6: Expected accuracy when predicting on 3 kbp phage sequences hosts over fewer orders. The error bars indicate 95% of confidence interval, over 300 replicates. For a better readability of the plot, the error bars are horizontally shifted by 0.1. Solid lines: WisH, dashed lines: VirHostMatcher.

4 Evaluation on 16S sequence identity

Yarza *et al.* (2014) state that "although there are official rules for the nomenclature of bacteria and archaea, the entities that are known as taxa and their hierarchical classifications are artificial constructs and so are somewhat subjective". As a consequence, bacteria of different genera can be very closely related whereas bacteria of the same genus can be more divergent. For example, certain *Escherichia* and *Shigella* strains have more than 99% residue identity for their 16S rRNA genes and certain *Escherichia* and *Salmonella*), whereas some bacteria in the same genus *Escherichia* have less than 96% residue identity among their 16S rRNA genes. Thus, a classification of bacteria and archaea based on a more quantitative criterion would be preferable in general and, in particular, to test the accuracy of the WIsH predictions. Yarza *et al.* (2014) proposes such a classification based on 16S sequence identity using 16S sequence identity thresholds that typically correspond to the usual taxonomy (these thresholds are reported in table 3). Tables 2 and 3 show the accuracies of WIsH and VHM under these 16S taxonomic criteria for 3kbp contigs and full-length genomes respectively.

Figure 7 shows the relation between the p-value of the best prediction made by WIsH and the 16S identity between the predicted host and the actual one.

Taxonomic level	Genus	Family	Order	Class	Phylum
WIsH accuracy – standard taxonomy (%)	32.3	36.8	42.1	55.9	70.1
VHM accuracy – standard taxonomy (%)	20.4	26.0	31.6	42.8	59.1
16S seq. id. threshold (%)	94.5	86.5	82.0	78.5	75.0
WIsH accuracy – 16S criterion (%)	38.4	55.5	63.8	69.3	87.1
VHM accuracy – 16S criterion (%)	26.1	46.1	55.3	63.8	82.1

Table 2: Accuracies of the predictions on the WIsH benchmark (3kbp) using 16S rRNA residue identity thresholds for taxonomic assignment.

Taxonomic level	Genus	Family	Order	Class	Phylum
WIsH accuracy – standard taxonomy (%)	42.5	47.6	51.6	63.0	76.7
VHM accuracy – standard taxonomy (%)	42.3	50.1	55.1	64.6	75.7
16S seq. id. threshold (%)	94.5	86.5	82.0	78.5	75.0
WIsH accuracy – 16S criterion (%)	46.9	62.6	70.4	74.7	89.5
VHM accuracy – 16S criterion (%)	50.1	65.9	73.5	77.3	87.6

Table 3: Accuracies of the predictions on the WIsH benchmark (full-length phage genomes) using 16S rRNA residue identity thresholds for taxonomic assignment.

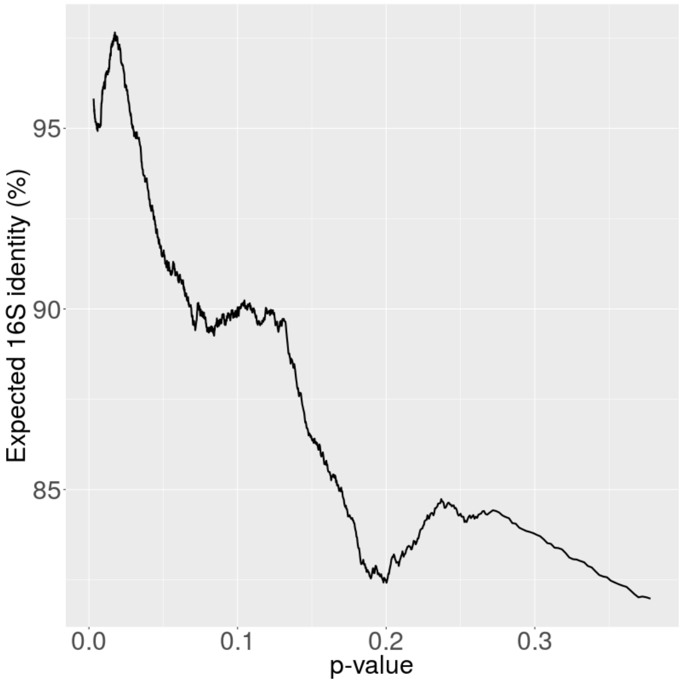

Figure 7: 16S percent identity between actual and predicted host with respect to the p-value threshold. The line is obtained by rolling-mean smoothing of the scatter plot with $k = 200$.

5 Evaluation with distant prokaryotic genomes

Since the diversity of the prokaryotic world is yet not fully known, the genomes of the actual host of a phage may still be unavailable. In this case, one shall rely on more distantly available genomes (organism in the same genus as the actual host for instance).

We evaluate here the accuracies of the prediction when removing from the evaluation the genomes of prokaryotes closely related to the actual host. More specifically, for a given phage, we predict only using models built on prokaryotes belonging to a different genus than the actual infected genus. We kept only the phages ($N = 1016$) infecting genus having more than a single genus in their family, since it cannot otherwise lead to any right prediction at family level. The resulting accuracies in table 4 do not prove to be enough for a reliable prediction of the taxonomy of the host. However, by restricting the number of possible orders in the prediction, as done in the section 3, we show on figure 8 that the prediction accuracies reach more acceptable values. WIsH and VirHostMatcher have comparable accuracy at family level, and WIsH improve on VirHost-Matcher on higher taxa prediction. VirHostMatcher is slightly better than WIsH at family level when testing on 10kbp contigs (cf. Figure 9), but the accuracy of both tools at family level is so low that it will not be useful in practice.

In practice, for each viral metagenome generated the prokaryotic fraction of the sample can be obtained and sequenced as well. By binning the prokaryotic metagenomic contigs, one can learn the WIsH models on the set of sequences of each bin (with model order that can be adjusted to the total length of the bins according to the graphs in section 2.3), and use them to scan the associated viral metagenome. If a virus is present in a significant amount, there is good hope for the infected organism to be also part of the prokaryotic fraction, and therefore to be accurately detected by WIsH.

Taxonomic level	Genus	Family	Order	Class	Phylum
WIsH accuracy – 16S criterion (%)	**10.8**	**39.5**	**51.8**	**60.9**	**81.6**
VHM accuracy – 16S criterion (%)	9.7	31.0	44.2	55.2	77.4
WIsH accuracy – std. taxonomy (%)	0	**13.8**	**28.0**	**48.8**	**62.6**
VHM accuracy – std. taxonomy (%)	0	12.7	22.1	38.4	51.0

Table 4: Accuracies of the predictions on the WIsH benchmark on 3kpb contigs when using only distantly related genomes from the host.

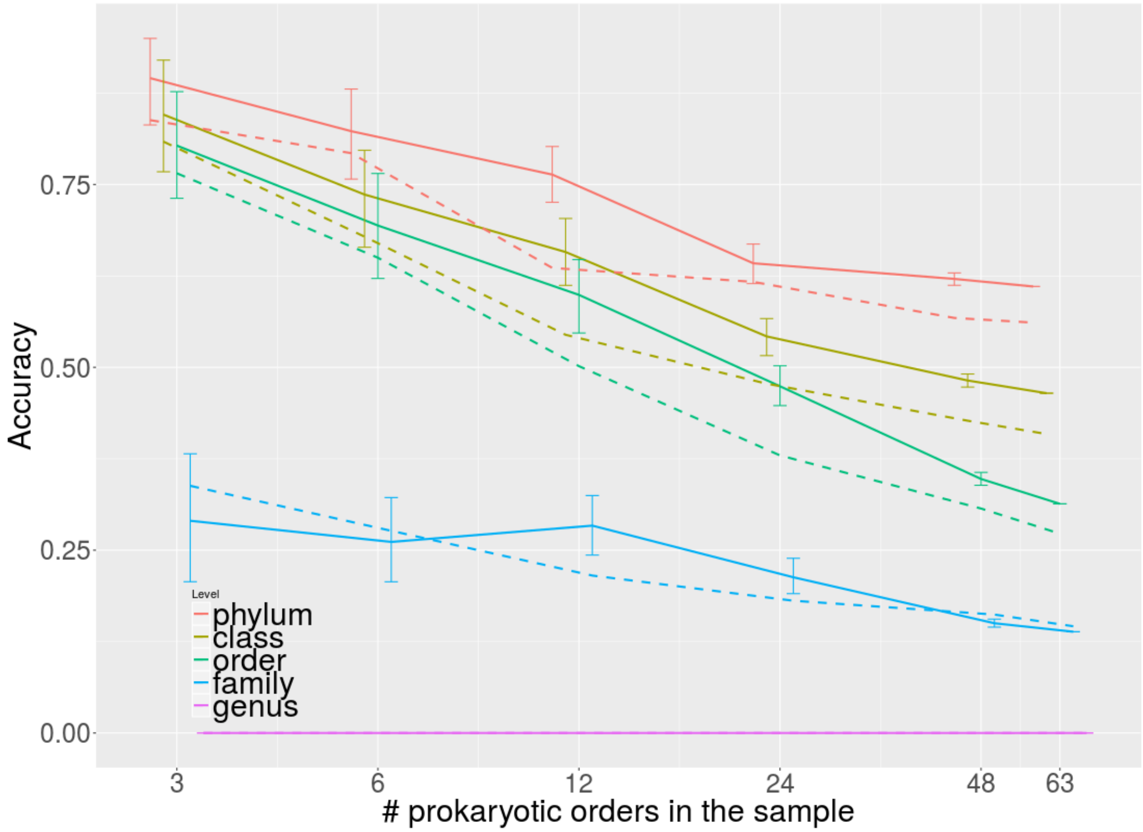

Figure 8: Accuracy of the predictions on 3kbp contigs when restrincting the diversity of the predictions and removing models corresponding to the infected genus. Solid lines: WIsH, dashed lines: VirHostMatcher.

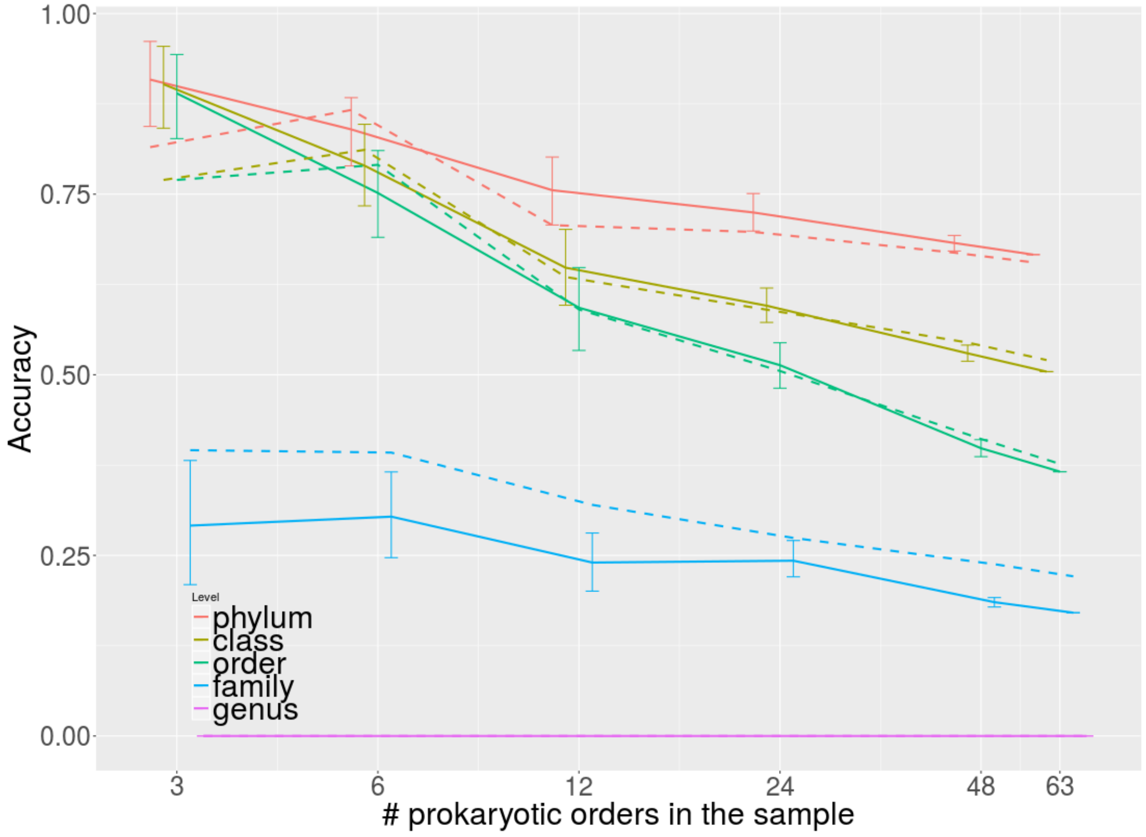

Figure 9: Accuracy of the predictions on 10kbp contigs when restrincting the diversity of the predictions and removing models corresponding to the infected genus. Solid lines: WIsH, dashed lines: VirHostMatcher.

6 Run times

Runtimes were measured on a Linux PC with two 8-core 2.6 GHz Intel Xeon E5-2640 v3 processors.

Dataset		WIsH	VirHostMatcher
Earth's virome contigs ($N = 125.842$)		10h39m26s	-
Contigs from WIsH benchmark ($N = 1420$)	1kb	**32s**	16h37m
	5kb	**2m20s**	16h44m
	10kb	**4m33s**	16h43m

Table 5: Runtimes for Earth's virome contigs and for genomic fragments of the pahges of the WIsH benchmark.

7 Annotation of Earth's virome contigs

Hosts have been predicted using WIsH for the 125,842 metagenomic viral contigs (mVCs) of the Earth's virome (Paez-Espino *et al.*, 2016) using prokaryotic models from the WIsH benchmark dataset. For a given p-value threshold on the prediction, the expected precision was measured – defined as the fraction of the WIsH predictions that match the original host annotation of (Paez-Espino *et al.*, 2016). These annotations originally covered 7.7% of the contigs using alternative host prediction methods such as CRISPR or tRNA sequence matches. Here, only the host annotations whose genus was represented in our prokaryotic dataset were kept, finally amounting to 5.3% of the mVCs. Figure 10 shows the expected precision with respect to the fraction of newly annotated contigs. For instance, setting a p-value threshold of 10^{-1} (red line) allows to find annotation for more than half of the unannotated contigs, while being consistent at 70% with the original host family annotation.

7.1 Factors influencing the prediction quality

7.2 Influence of encoded tRNAs

The more tRNA encoded in the viral genome, the worse was the prediction. The number of true (in green) and wrong (in red) predictions at species and genus level respectively are plotted in figures 11 and 12. At species level, all predictions made for phage genomes encoding more than 4 tRNAs are wrong. This is also significant at genus level since the 10 correctly predicted phages encoding more than 30 tRNA in figure 12 come down to only 1 single phage infecting the genus *Mycobacterium* (the 10 instances are all coming from the cluster C1 described in Pope *et al.* (2015)).

Taxonomic level	Specie	Genus	Family	Order	Class	Phylum
p-value	5.10^{-12}	3.10^{-11}	9.10^{-16}	2.10^{-16}	2.10^{-11}	5.10^{-11}

Table 6: p-value under a Wilcoxon rank-sum test of number of encoded tRNAs between phages with correctly and wrongly predicted host.

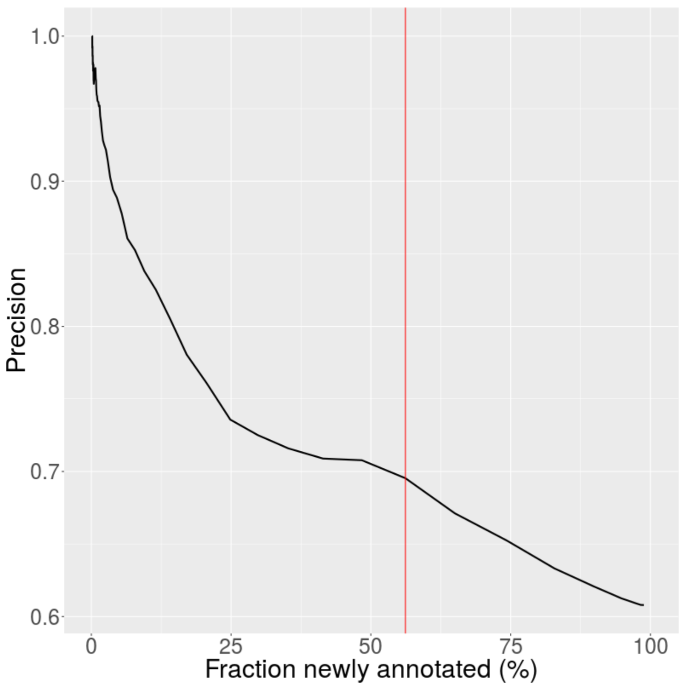

Figure 10: Precision (using original annotation as reference) at family level vs. fraction of newly annotated contigs.

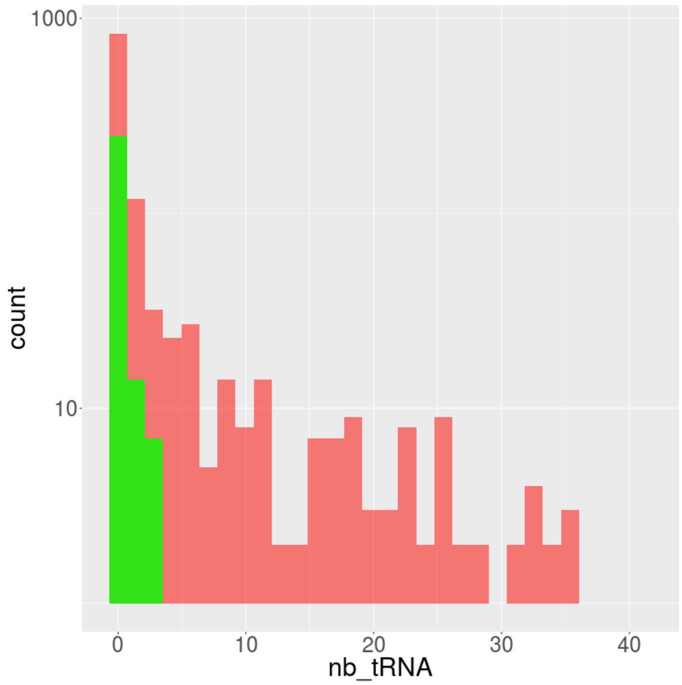

Figure 11: Distribution of the number of encoded tRNAs for correct (green) or wrong (red) host predictions for 1,420 phages of the WIsH benchmark, at **species** level.

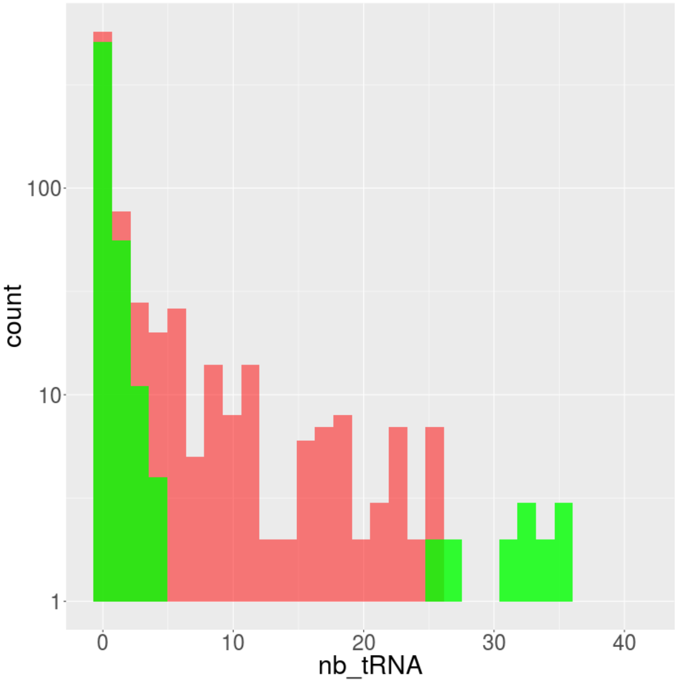

Figure 12: Distribution of the number of encoded tRNAs for correct (green) or wrong (red) host predictions for 1,420 phages of the WIsH benchmark, at **genus** level.

7.3 Influence of number of coding sequences on prediction

As for the number of encoded tRNAs, the more coding sequences (CDS) present in the genome, the less accurate the host prediction gets.

Taxonomic level	Specie	Genus	Family	Order	Class	Phylum
p-value	1.10^{-12}	1.10^{-5}	9.10^{-9}	1.10^{-7}	3.10^{-7}	1.10^{-7}

Table 7: p-value under a Wilcoxon rank-sum test of number of coding sequences between phages with correctly and wrongly predicted host.

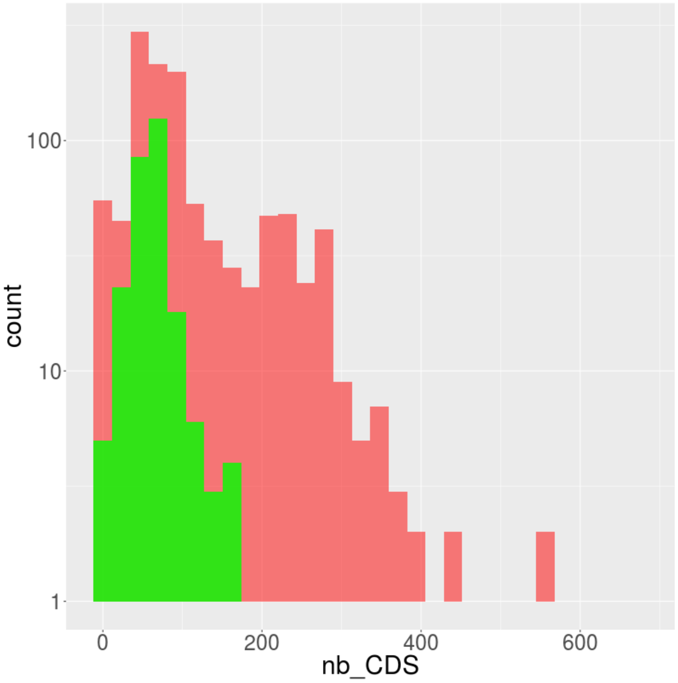

Figure 13: Distribution of the number of CDS for correct (green) or wrong (red) best host predictions for 1,420 phages of the WIsH benchmark, at **species** level.

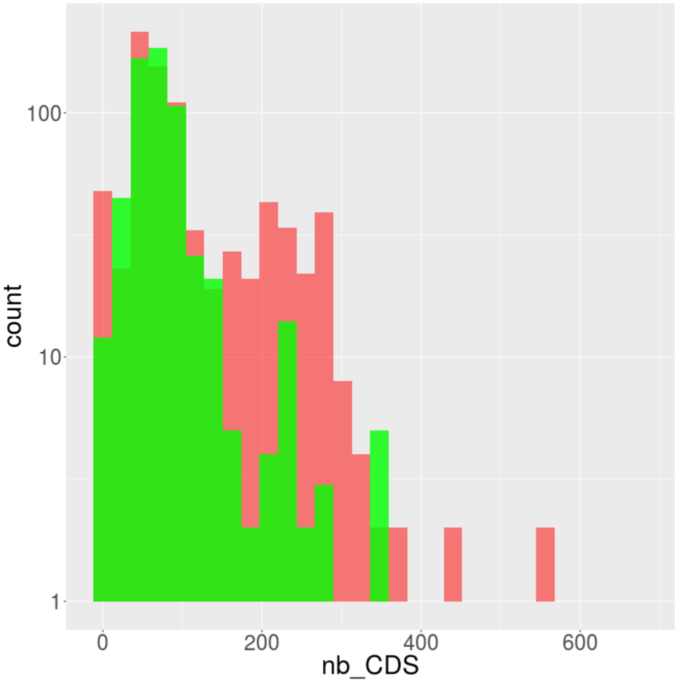

Figure 14: Distribution of the number of CDS for correct (green) or wrong (red) best host predictions for 1,420 phages of the WIsH benchmark, at **genus** level.

References

Ahlgren, N. A., Ren, J., Lu, Y. Y., Fuhrman, J. A., and Sun, F. (2016). Alignment-free d_2^* oligonucleotide frequency dissimilarity measure improves prediction of hosts from metagenomically-derived viral sequences. *Nucleic Acids Research*, **45**(1), 39.

França, L., Sannino, C., Turchetti, B., Buzzini, P., and Margesin, R. (2016). Seasonal and altitudinal changes of culturable bacterial and yeast diversity in alpine forest soils. *Extremophiles*, **20**(6), 855–873.

Nasidze, I., Li, J., Quinque, D., Tang, K., and Stoneking, M. (2009). Global diversity in the human salivary microbiome. *Genome research*, **19**(4), 636–643.

Paez-Espino, D., Eloe-Fadrosh, E. A., Pavlopoulos, G. A., Thomas, A. D., Huntemann, M., Mikhailova, N., Rubin, E., Ivanova, N. N., and Kyrpides, N. C. (2016). Uncovering earth's virome. *Nature*, **536**(7617), 425–430.

Pope, W. H., Bowman, C. A., Russell, D. A., Jacobs-Sera, D., Asai, D. J., Cresawn, S. G., Jacobs Jr, W. R., Hendrix, R. W., Lawrence, J. G., Hatfull, G. F., *et al.* (2015). Whole genome comparison of a large collection of mycobacteriophages reveals a continuum of phage genetic diversity. *Elife*, **4**, e06416.

Santigli, E., Trajanoski, S., Eberhard, K., and Klug, B. (2016). Sampling modification effects in the subgingival microbiome profile of healthy children. *Frontiers in Microbiology*, **7**, 2142.

Yarza, P., Yilmaz, P., Pruesse, E., Glöckner, F. O., Ludwig, W., Schleifer, K.-H., Whitman, W. B., Euzéby, J., Amann, R., and Rosselló-Móra, R. (2014). Uniting the classification of cultured and uncultured bacteria and archaea using 16s rrna gene sequences. *Nature Reviews Microbiology*, **12**(9), 635–645.
